# Systematic identification of reference genes for qRT-PCR of *Ardisia kteniophylla* A. DC under different experimental conditions and for anthocyanin-related genes studies

**DOI:** 10.3389/fpls.2023.1284007

**Published:** 2023-11-02

**Authors:** Wentao Wang, Xiaohang Zhang, Xiaoxia Xu, Xingchou Xu, Lin Fu, Hongfeng Chen

**Affiliations:** ^1^ Key Laboratory of Plant Resources Conservation and Sustainable Utilization, South China Botanical Garden, Chinese Academy of Sciences, Guangzhou, China; ^2^ College of Modern Agricultural Sciences, University of Chinese Acadamy of Science, Beijing, China; ^3^ College of Life Science, Gannan Normal University, Ganzhou, China

**Keywords:** *Ardisia kteniophylla* A. DC, qRT-PCR, gene expression, reference gene, different experimental conditions

## Abstract

*Ardisia kteniophylla* A. DC, widely known as folk medicinal herb and ornamental plant, has been extensively investigated due to its unique leaf color, anti-cancer and other pharmacological activities. The quantitative real-time PCR (qRT-PCR) was an excellent tool for the analysis of gene expression with its high sensitivity and quantitative properties. Normalizing gene expression with stable reference genes was essential for qRT-PCR accuracy. In addition, no studies have yet been performed on the selection, verification and stability of internal reference genes suitable for *A. kteniophylla*, which has greatly hindered the biomolecular researches of this species. In this study, 29 candidate genes were successfully screened according to stable expression patterns of large-scale RNA seq data that from a variety of tissues and the roots of different growth stages in *A. kteniophylla*. The candidates were then further determined via qRT-PCR in various experimental samples, including MeJA, ABA, SA, NaCl, CuSO_4_, AgNO_3_, MnSO_4_, CoCl_2_, drought, low temperature, heat, waterlogging, wounding and oxidative stress. To assess the stability of the candidates, five commonly used strategies were employed: delta-CT, geNorm, BestKeeper, NormFinder, and the comprehensive tool RefFinder. In summary, *UBC2* and *UBA1* were found to be effective in accurately normalizing target gene expression in *A. kteniophella* regardless of experimental conditions, while *PP2A-2* had the lowest stability. Additionally, to verify the reliability of the recommended reference genes under different colored leaf samples, we examined the expression patterns of six genes associated with anthocyanin synthesis and regulation. Our findings suggested that *PAP1* and *ANS3* may be involved in leaf color change in *A. kteniphella*. This study successfully identified the ideal reference gene for qRT-PCR analysis in *A. kteniphella*, providing a foundation for future research on gene function, particularly in the biosynthesis of anthocyanins.

## Introduction

*Ardisia kteniophylla* A. DC, an evergreen shrub from the genus *Ardisia* (*Primulaceae* family), was mainly found in South China, North Vietnam, Laos and Thailand, and commonly known as a traditional folk medicine to treat rheumatism and injuries from muscles and fractures in China (Guan et al., 2016; Huang et al., 2017). It was first documented in the classical medical book “Sheng Cao Yao Xing Bei Yao” in the Qing dynasty (written in 1711), generally utilized by ethnic groups such as Yao, Zhuang, Dong, Jing, Li, Miao (Tian et al., 2023). Recently, phytochemical studies have found various chemical components that mainly contained triterpenoids, coumarins, flavonoids, phenols, quinones, sterols, and volatile oils, which active in a wide variety of biological processes such as anti-cancer, anti-HIV, anti-thrombotic anti-infammation, anti-oxidant, and cough expectorant activities(Mu et al., 2015; Mu et al., 2017; Mu et al., 2012; Tian et al., 2023). In addition, in the seedling stage, this plant is also served as an ornamental plant for its leaves are brightly colored, including red, semi red, and green(Tian et al., 2023; Zhao et al., 2021). Current research indicates that the biosynthetic pathways of anthocyanins have been identified in other plants, and found that anthocyanidin synthase (ANS) catalyzes leucocyanidin and catechin for conversion to cyanidin (Jun et al., 2018). Additionally, transcription factors PAP1 often regulate structural genes in the anthocyanin pathway in other plants, including strawberry (Zhang et al., 2020), carrot (Xu et al., 2020), tomato (Liu et al., 2016), and apple (An et al., 2020). Thus, ANS and PAP1 can regulate the expression of anthocyanin biosynthesis genes to influence leaf color. However, the molecular mechanisms involved in the color formation and transformation of leaves in *A. kteniophylla* are yet to be fully understood. Gene expression analysis has so far been a foundation for understanding gene function and molecular mechanisms in the development of organisms.

Gene expression analysis was critical approach for revealing gene function and enhancing understanding understanding the molecular mechanisms underlying diverse plant biological processes. At present, a variety of methods were available for assessing gene expression, including northern blotting, *in situ* hybridization, gene chips, site-directed mutagenesis, and qRT-PCR (Gao et al., 2017; Wang et al., 2021; Yang et al., 2020). Among them, qRT-PCR, a particularly popular and effective method, has gained wide acceptance for its high sensitivity, accuracy, reliability, and specificity for detecting gene expression (Pfaffl et al., 2004). The reliability of gene expression measurements via qRT-PCR analysis, however, was strongly influenced by several factors, including RNA recovery and integrity, PCR amplification, PCR efficiency, primer design and the sample transcriptional activity (Pfaffl et al., 2004; Udvardi et al., 2008; Wang et al., 2021; Yang et al., 2020). Thus, to dispel these negative influences, technical differences from different samples and experimental environments were minimized by normalizing transcript levels of stable reference genes. The search for ideal reference genes that can be expressed under a variety of experimental conditions has been undertaken by several researchers. Unfortunately, most of these reference genes are not universally applicable to all plant species (Luo et al., 2018; Nolan et al., 2006; Udvardi et al., 2008; Wang et al., 2016). Therefore, a systematized approach to identify and evaluate multiple stable reference genes in *A. kteniophylla* was necessary to perform accurate transcript normalization experiments.

In recent years, Next Generation Sequencing (NGS) has been widely used in fields such as genome sequencing, RNA seq, ChIP sequencing, and has become a commonly used biotechnology for its high resolution, low cost, and high sensitivity (de Magalhães et al., 2010; Stone and Storchova, 2015). Most recently, RNA-seq data has been extensively utilized to identify new reference genes in plants, thereby serving as a potential source of candidate genes for various plant species, such as soybean (Yim et al., 2015), grape (González-Agüero et al., 2013), *Lycoris aurea* (Ma et al., 2016), *Alternanthera philoxeroides* (Yin et al., 2021), *Zingiber officinale* (Li et al., 2022) and apple (Zhou et al., 2017). To screen the stable reference genes, four frequently statistical processing tools: geNorm (Vandesompele et al., 2002), NormFinder (Andersen et al., 2004), BestKeeper (Pfaffl et al., 2004), and ΔCt methods (Silver et al., 2006), were commonly used to evaluate the best suitable candidate, which were then analyzed from different perspectives. In addition, RefFinder, as a web-based comprehensive analytics tool, which can combine all four of these methods to compare and rank the optimal candidate reference genes (Xie et al., 2012). Consequently, these statistical software have been successfully applied to selecting the reference genes from a range of candidate genes in various plants, such as *Codonopsis pilosula* (Yang et al., 2020), *Scutellaria baicalensis* (Wang et al., 2021), *Allium sativum* (Wang et al., 2023), and so on. However, no systematic studies have been done on *A. kteniophylla* reference genes for qRT-PCR standardization, which greatly limits the research of functional genes in *A. kteniophylla*.

Herein, we used a comprehensive expression profile of large-scale RNAseq data that from a variety of different tissues and the roots of different growth stages to select for the most stable genes expressed in *A. kteniophylla*. Subsequently, 29 candidate genes were successfully screened and their expression levels were further determined via qRT-PCR in 14 experimental samples. Finally, five commonly used statistical algorithms—delta-CT, geNorm, BestKeeper, NormFinder, and the comprehensive tool RefFinder were used to determine the stability of those candidates and rank them. Furthermore, to confirm the reliability and applicability of the reference genes, we analyzed the expression patterns of target genes related to anthocyanin regulation and synthesis. This work represents the initial systematic investigation of stable reference genes in *A. kteniophylla*, serving as a fundamental basis for future research on the molecular mechanisms underlying biological functions in *A. kteniophylla*, with a particular focus on the expression of genes related to anthocyanin synthesis.

## Materials and methods

### Selecting reference genes for candidate analysis

An investigation was conducted to determine the gene expression patterns of *A. kteniophylla* utilizing RNA-seq data from various tissues, such as mainroot, fibrous root, root nodule, stem xylem, stem phloem, leaf, flower, and fruit, as well as roots of varying growth stages, including 1 month old, 3 months old, 6 months old, 1 year old, 2 years old, and 3 years old, which has yet to be published by our laboratory. A total of 42 data sets were used to calculate fragments per kilobase per million reads (FPKM), mean values (MV) and standard deviations (SD) for each gene. To identify genes with high expression levels and stability, we selected genes with MV values greater than 30 and ranked them based on their SD values from the smallest to the highest. The top 500 genes were further filtered on the basis of functional annotations to identify 29 candidates with commonly known functions. The heatmap were generated using TBtools software (Chen et al., 2020), and box plot using GraphPad Prism 7 software (Mitteer et al., 2018). Furthermore, the computation of the coefficient of variation (CV, FPKM/mean FPKM) and the maximum coefficient of variation (MFC; Maximum FPKM value/minimum FPKM value) was performed and utilized to generate dot plots for evaluating the expression stability of the 29 chosen genes, following the methodology described in the literature (Yin et al., 2021).

### Plant materials and stress treatments

*A. kteniophylla* seeds were gathered from Nanxiong City, Guangdong Province, China. They were planted in pots (3 plants per pot) filled with soil mixture (peat: perlite = 1:1) and grown in the greenhouse of South China Botanical Garden, Chinese Academy of Sciences (Guangzhou, Guangdong Province, China, 112.3571°E, 23.1772°N). Healthy six-month-old seedlings were divided into different groups and subsequently receive abiotic stress and hormone treatments.

A total of fourteen experimental groups were performed: including 100 μmol MeJA (methyl jasmonate, 12h), 100 mM ABA (abscisic acid, 12h), 100 μmol SA (salicylic acid, 12h), 100 mmol NaCl (Na, 12h), 200 µM CuSO_4_ (Cu, 12h), 1 mmol AgNO_3_ (Ag, 12h), 200 μmol MnSO_4_ (Mn, 12h), 100 μmol CoCl_2_ (Co, 12h), Drought (20% PEG6000), low temperature (4°C, 24h), heat stress (35°C, 24h),Waterlogging (sinking 1-2cm underwater), Wounding (Blade scratches about 50% of plant leaves, 12h) and Oxidative stress (treated with 50 mM H_2_O_2_, 24 h). All control and experimental plants had three biological replicates. Prior to RNA extraction, the samples were gathered and frozen immediately in liquid nitrogen, then stored at -80°C.

### Total RNA extraction and cDNA synthesis

The Fastpure® Universal Plant Total RNA Isolation Kit (Vazyme, Nanjing, China) was utilized to extract total RNAs from each collected sample in accordance with the recommended protocols of the supplier. Subsequently, the integrity and quality of the total RNA samples were assessed through 1 g L^−1^ agarose gel electrophoresis, while the concentration and purity of RNAs were determined using a NanoDrop 2000c spectrophotometer (Thermo Fisher Scientific, Waltham, MA, USA). The RNAs were deemed acceptable if they exhibited an optical density of A260/280 ≈ 1.8–2.1 and A260/230 ≈ 1.7–2.2. The HiScript®II Reverse Reverse Transcriptase (Vazyme, Nanjing, China) was employed to synthesize single-strand cDNA from 1.0 µg of total RNA in a 20 µL volume system, following the manufacturer’s protocols. Prior to use, all cDNA samples were diluted with nuclease-free water (1:20) and stored at -20°C.

### Primer design and qRT-PCR

The gene-specific primers utilized for qRT-PCR (as presented in **Table 1**) were developed through the utilization of Primer Premier 5.0 (Premier, Inc., Toronto, Canada), with the following parameters: an amplicon length of 80-200 bp, primer length ranging from 18-22 bp, a melting temperature (Tm) of 58-61°C, and a GC content of 50-66%. The primers were subsequently synthesized by Tsingke Biotechnology Co., Ltd (Beijing, China). The primer pair specificity and amplicon size was tested by melting curve analysis and gel electrophoresis, PCR efficiency (*E*) and regression coefficient (*R^2^*) were assessed using the diluted cDNA, as described earlier (Kudo et al., 2016). LightCycler® 480 System (Roche Molecular Systems, Germany) was used for qRT-PCR using LightCycler® 384 Plates. The reaction system were 10 μL in volume and contained 1 µL 20-fold diluted cDNA, 5 µL of 2 ×Vazyme qPCR SYBR Green Master Mix (Vazyme,Nanjing, China), 0.5µL of each forward and reverse primer and ddH_2_O 3.5 µL. The cycling conditions were initiated with an initial temperature of 95°C for 30 s, followed by 40 cycles of 95°C for 5 s and 60°C for 30 s, and concluded with a final melt-curve analysis. Analytical replicates of all qRT-PCR experiments were conducted three times.

**Table 1:** Details of candidate reference genes and validation genes for qRT-PCR normalization in *A. kteniophylla*.

Gene symbol	Description	Gene_ID	Primer Sequence	Primer Tm	Amplicon size (bp)	Slope	*R²*	Amplification efficiency (%)
***PP2A-1***	Serine/threonine-protein phosphatase 2A 65 kDa	evm.TU.Chr20.651	F:TCGCACCCTTGGAAACCC	59.9	142	3.3887	0.9992	97.29
			R:GCTGCCAGCCTCTTGACA	60.0				
***PP2A-2***	Serine/threonine-protein phosphatase PP2A-2	evm.TU.Chr19.1077	F:GGGGCCAATGTGCGATCT	60.1	81	3.2503	0.995	103.08
			R:GTGTATCCAGCGCCCCTG	60.2				
***UBA1***	Ubiquitin-activating enzyme E1 1	evm.TU.Chr3.1901	F:GTTTGACCGCCCACCTCT	60.4	96	3.4052	0.9964	96.64
			R:TCCTCTTCCGAGCCAGCA	60.2				
***UBC35***	Ubiquitin-conjugating enzyme E2 35	evm.TU.Chr5.2175	F:GGCTCCCAAGGTTCGGTT	59.6	101	3.3897	0.9986	97.25
			R:GGGCAGGGCTCCATTTGT	60.0				
***UBC9***	Ubiquitin-conjugating enzyme E2 9	evm.TU.Chr19.2697	F:TAGTCCTTATACTGGCGGTGTC	59.4	124	3.3533	0.9942	98.71
			R:GCTCCCGTTGCTGTTGAT	58.6				
***UBC5***	Ubiquitin-conjugating enzyme 5	evm.TU.Chr15.2454	F:GCGGGACCGAACTGCTTA	59.7	150	3.3745	0.9964	97.85
			R:CCCGCTACTGCCTCATCG	60.0				
***UBC2***	Ubiquitin-conjugating enzyme 2	evm.TU.Chr14.1266	F:GTCCGATCTACGATGTGGC	58.3	128	3.3247	0.9901	99.88
			R:TCTGTTGTATTCCCGCTTG	59.5				
***Actin-3***	Actin-3	evm.TU.Chr13.2655	F:TGATGATGCTCCACGGGC	59.8	80	3.3131	0.9994	100.37
			R:TCTGACCCATCCCGACCA	59.6				
***Actin-7***	Actin-7	evm.TU.Chr13.2335	F:ATCCTCCGTCTGGACCTTG	58.7	102	3.3373	0.9968	99.36
			R:AATTTCCCGTTCTGCTGTG	58.0				
***ADF-2***	Actin-depolymerizing factor 2	evm.TU.Chr19.676	F:CTGCACGTGTGAGGAGCA	60.0	88	3.4725	0.9998	94.08
			R:GCTTGCAGCTCCACCTGA	60.0				
***GAPDH-2***	Glyceraldehyde-3-phosphate dehydrogenase 2	evm.TU.Chr6.381	F:TCGGAAGGATCGGACGGT	60.0	148	3.3306	0.9993	99.64
			R:TCACTGTGCTTCCAGCGG	60.0				
***GAPDH-1***	Glyceraldehyde-3-phosphate dehydrogenase	evm.TU.Chr23.3126	F:TGCCACGCAGAAGACTGT	59.2	110	3.3546	0.9916	98.65
			R:TCCCAACAGCCTTGGCAG	59.9				
***CYP21***	Peptidyl-prolyl cis-trans isomerase CYP21-4	evm.TU.Chr3.2731	F:AAACCTCAGGCGCTGCTT	59.9	146	3.4389	0.9989	95.34
			R:TGTGACCTCCGAGCCCAA	60.5				
***CYP1***	Peptidyl-prolyl cis-trans isomerase 1	evm.TU.Chr14.3084	F:GGGCGGATCGTGATGGAG	60.0	137	3.3217	0.9913	100.01
			R:ACGCGGTGGAACTTGGAG	60.0				
***EF-1-α -1***	Elongation factor 1-alpha 1	evm.TU.Chr21.3815	F:GGCCCAACCCTCCTTGAC	60.0	80	3.4712	0.9921	94.13
			R:TGGAGTGGGAGACGGAGG	60.0				
***EF-1-α-2***	Elongation factor 1-alpha	evm.TU.Chr12.240	F:AAGGGCCCCACATTGCTC	60.0	137	3.3183	0.9993	100.15
			R:GTCTCAACACGGCCGACA	60.0				
***β-TUB-1***	Tubulin beta-1 chain	evm.TU.Chr6.1831	F:AGTGGGGTCACATGCTGC	60.0	90	3.349	0.9992	98.88
			R:GAGACGGGGGAAGGGGAT	60.0				
***β-TUB-2***	Tubulin beta-2 chain	evm.TU.Chr6.1665	F:CCCCTTCCCCCGTCTACA	60.0	90	3.2351	0.9951	103.76
			R:TCCGGGACTGTGAGGGAG	60.0				
***α-TUB-3***	Tubulin alpha-3 chain	evm.TU.Chr21.3206	F:CTGCTGTGGCCACCATCA	60.0	118	3.3079	0.9836	100.59
			R:GCTAGATCACCACCCGGC	59.9				
***α-TUB-4***	Tubulin alpha-4 chain	evm.TU.Chr13.137	F:ACCTCTGTGGTTGAGCCTTAC	59.6	157	3.321	0.9991	100.04
			R:CCAGCCTGTTGAGATTAGTGTA	57.6				
***RPL13***	60S ribosomal protein L13-1	evm.TU.Chr19.2535	F:GTCATCCCGAATGGGCACT	59.7	151	3.3075	0.9849	100.61
			R:GGACAATGGGACGGAGTTT	58.6				
***HSP70***	Heat shock cognate 70 kDa protein	evm.TU.Chr13.1922	F:CTCAGACAACCAGCCGGG	60.0	103	3.3274	0.9985	99.77
			R:GGGAATGCCCGAGAGCTC	59.9				
***HSP90***	Heat shock cognate protein 90	evm.TU.Chr18.137	F:TCAGCCTCGATGACCCCA	60.0	97	3.342	0.9933	99.17
			R:TCCGCATCAGCATCAGCC	60.2				
***ATP***	ATP synthase subunit O	evm.TU.Chr9.1309	F:CGATGGCTGGGCGTATGA	59.6	103	3.2106	0.9914	104.87
			R:TTAGCGAAGGTGGGGCAC	59.7				
***SAM5***	S-adenosylmethionine synthase 5	evm.TU.Chr7.30	F:TCAATATCGAGCAGCAGAGC	57.8	114	3.2452	0.9964	103.30
			R:CGGTGGCATAACCAAACAT	56.1				
***SAM2***	S-adenosylmethionine synthase 2	evm.TU.Chr19.1139	F:GGGTACGCCACTGACGAGA	61.9	179	3.2324	0.9934	103.88
			R:CGCACAGGGACCATAGCAC	61.3				
***26S***	26S proteasome non-ATPase	evm.TU.Chr9.374	F:ACCGCCAGGATTACGTGC	60.1	127	3.2378	0.9947	103.63
			R:GCTGGAGCCTCTTCCACG	60.1				
***30S***	40S ribosomal protein S30	evm.TU.Chr7.1634	F:GACACCCAAGGTAGCCAAGC	59.5	96	3.2976	0.9913	101.03
			R:ACAACGGCAGTGACGAAGC	59.1				
***EIF-1A***	Eukaryotic translation initiation factor 1A	evm.TU.Chr5.1334	F:GAGGCCGACGACGAGAAG	59.9	127	3.3032	0.9999	100.79
			R:GACAAAGCCGCTTGGTGC	60.1				
***PAP1***	PRODUCTIO OF ANTHOCYANIN PIGMENT1 (PAP1)	evm.TU.Chr12.1675	CAACCTTTTCCGGGGTCGA	59.93	111	3.3032	0.9999	100.79
			GCATGCTATCAGGCCACCA	60.15				
***TT8***	TRANSPARENT TESTA8 (TT8)	evm.TU.Chr3.2529	CCAAAAGGCGAGGGCTAAG	60.10	167	3.3603	0.9926	98.42
			GTGGTGGAGGGGGAGGAGA	61.50				
***ANS1***	Anthocyanidin synthase1	evm.TU.Chr1.291	AGTACGCGAACGACCACG	60.13	118	3.3156	0.9919	100.26
			TCCTCCGGGAAGACGAGG	60.04				
***ANS2***	Anthocyanidin synthase2	evm.TU.Chr1.883	ACCCGAAATGCCCTCAGC	60.05	81	3.338	0.9942	99.33
			CGGGGAGGAGAAGGGTCA	59.96				
***ANS3***	Anthocyanidin synthase3	evm.TU.Chr16.613	ACGCTTGGGCTATCGTCG	59.90	118	3.3533	0.9968	98.71
			GGGCGGGTTTAACGGTGA	59.97				
***ANS4***	Anthocyanidin synthase4	evm.TU.Chr19.3002	ACCAGCTCCGCATGCTTT	59.97	89	3.3239	0.9913	99.92
			TGGCTCTGTGCTCCAAGC	59.97				

### Assessment of expression stability

To assess the stability of the 29 potential reference genes, five analytical tools, namely the Delta Ct method, BestKeeper, geNorm, Normfinder, and RefFinder, were utilized to determine the stabilities of each gene across varying experimental conditions (Andersen et al., 2004; Pfaffl et al., 2004; Rhinn et al., 2008; Silver et al., 2006; Vandesompele et al., 2002). Among them, the Delta Ct method enabled screening of appropriate references based on the SD value of Ct values, of which the smaller SD value indicate a higher gene stability (Andersen et al., 2004). The BestKeeper can determine the stabilization of the references via the coefficient of variation (CV) and standard deviation (SD) calculated based on the Ct values, with higher stability associated with lower CV and SD values (Pfaffl et al., 2004). GeNorm calculates an *M*-value based on the Ct value, with lower *M*-values represented greater stability (Rhinn et al., 2008). The Normfinder is an ANOVA-based model approach which calculates the SV value to assess the expression stability, where the lower SV value indicates a higher expression stability (Silver et al., 2006). As a final step, RefFinder, as a comprehensive tool (Vandesompele et al., 2002), computed the geometric average of the weights assigned to each gene in accordance with the four methods worked out previously.

### Stability verification of reference genes

The leaves of *A. kteniophylla* seedling show different colors including red, semi-red and green for the different anthocyanin content. Here, to assess the stability of the potential suitable reference genes, three sample sets were prepared, including samples of red, semi-red, and green leaf of *A. kteniophylla* seedling. Each sample was immediately frozen in liquid nitrogen and stored at a temperature of −80°C after being collected. The 2^-△△Ct^ method was used to determine the relative expression of investigated genes (Kumar et al., 2011). For the quantification of double reference genes, △△Ct was calculated as [((Ct gene – Ct reference 1) experiment – (Ct gene –Ct reference1) control)/2 + ((Ct gene – Ct reference 2) experiment– (Ct gene – Ct reference2) control)/2] (Gou et al., 2020).

## Results

### RNA-seq data selection for candidate reference genes in *A. kteniophylla*

**Figure 1:**
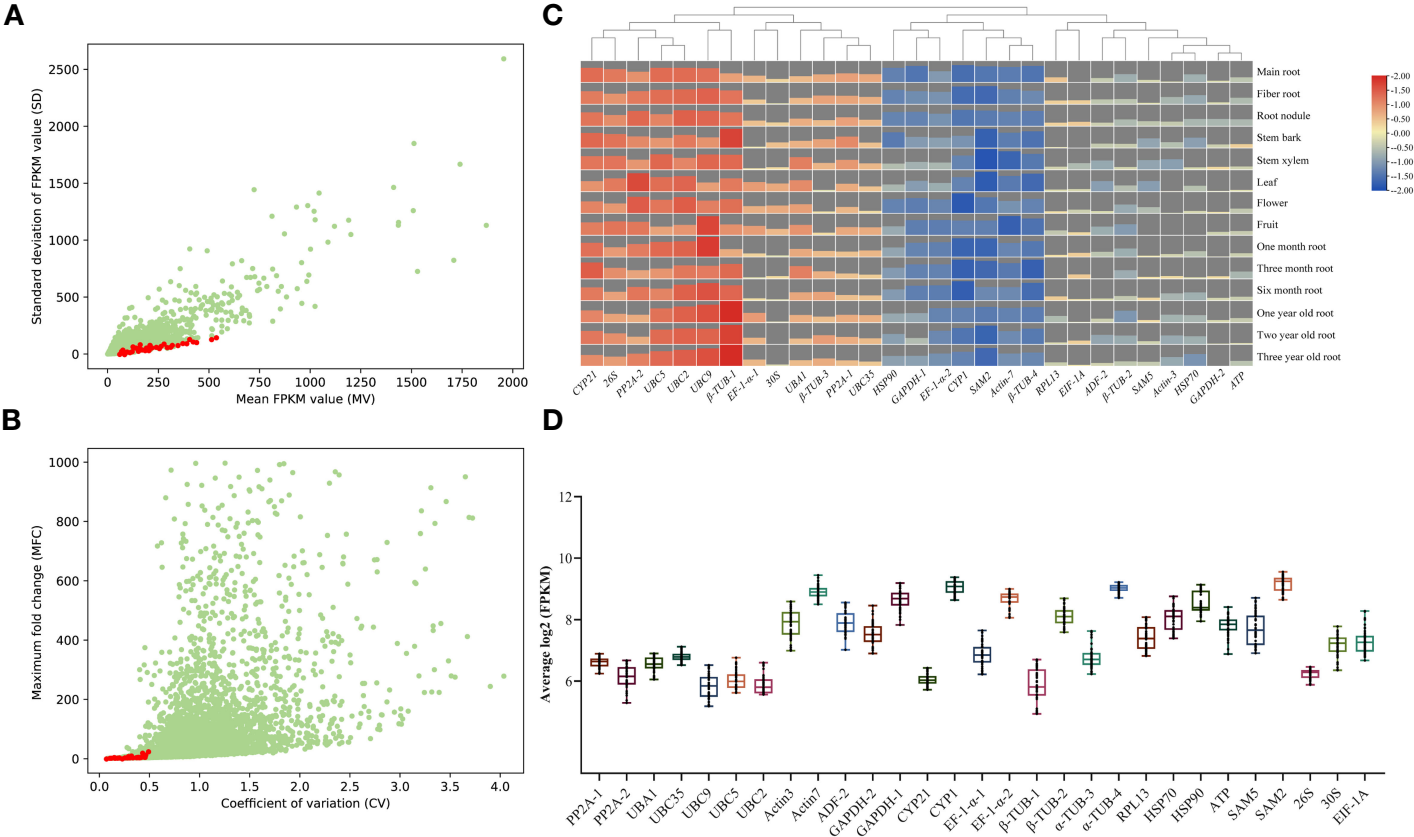
RNA-seq data selection for candidate reference genes in *A. kteniophylla*. The transcriptome’s candidate genes are presented through dot plots that display their mean (FPKM) and standard deviation (SD) values **(A)**, as well as their coefficient of variation (CV) and maximum fold change (MFC) **(B)**. **(C)** The expression levels of 29 candidate genes across various tissues and growth years are depicted in the heat map. The bar charts represent the expression levels of corresponding genes across various stages and tissues. A higher bar chart indicates a higher level of expression, while a lower bar chart indicates a lower level of expression. **(D)** Overview of the expression levels of 29 potential reference genes is provided through box and whisker plot graphs that exhibit the log2 (FPKM) values of each selected gene in multiple tissues. The 25th and 75th percentiles and medians are represented by black boxes and lines, respectively. Whisker caps represent the maximum and minimum values.

In order to select suitable candidate references, RNA-seq was conducted to assess gene expression levels in *A. kteniophylla*, which were subsequently normalized by FPKM values. The FPKM value was then utilized to calculate the mean value (MV) and standard deviation (SD). The candidate genes were arranged in ascending order based on their SD, and 29 genes with MV exceeding 30 and possessing functional annotations were chosen (**Figure 1A** and **Supplementary Table 1**). In order to conduct a more comprehensive assessment of the expression stability of the genes in question, the Mean Fold Change (MFC) and Coefficient of Variation (CV) were computed. As depicted in **Figure 1B** and **Supplementary Table 1**, the MFC and CV values for the 29 candidate genes were found to be less than 6.3 and 0.5, respectively. Taken together, the aforementioned four evaluation metrics (i.e., MV, SD, MFC, and CV values) collectively suggest that the 29 selected candidate genes are suitable for subsequent screening as ideal reference genes. To further investigate the stability of expression of 29 chosen genes, heat maps (**Figure 1C** and **Supplementary Table 2**) and box plots (**Figure 1D**) were generated using RNA-seq data. The results demonstrate that the expression of these genes remained remarkably stable and consistent across various growth stages and tissues. Therefore, it can be inferred that the selected genes are dependable and trustworthy.

### Primer specifcity and PCR efficiency analysis of reference gene

In order to identify optimal reference genes for gene expression profiling of *A. kteniophylla*, primers for 29 commonly utilized reference genes in qRT-PCR were designed using Primer Premier 5 (**Supplementary Table 3**). The specificity of these primer pairs was subsequently confirmed via electrophoresis on agarose gels, which demonstrated that each reference gene was specifically amplified and presented as a singular band, as anticipated (**Supplementary Figure 1**). Furthermore, melting curve analysis revealed that each amplicon exhibited only one peak (**Supplementary Figure 2**). The findings indicate that the primers exhibited a high degree of specificity and were suitable for gene expression analysis via qRT-PCR. The PCR amplification efficiency (*E*) was assessed and the results, presented in **Supplementary Figure 3** and **Table 1**, revealed that the *E* values for each primer pair ranged from 94.08% (*ADF*) to 104.87% (*ATP*). Additionally, the regression coefficient (*R^2^*) values varied between 0.9836 for *α-TUB-3* and 0.9999 for *EIF-1A*.

### Analyzing reference gene stability comprehensively

**Figure 2:**
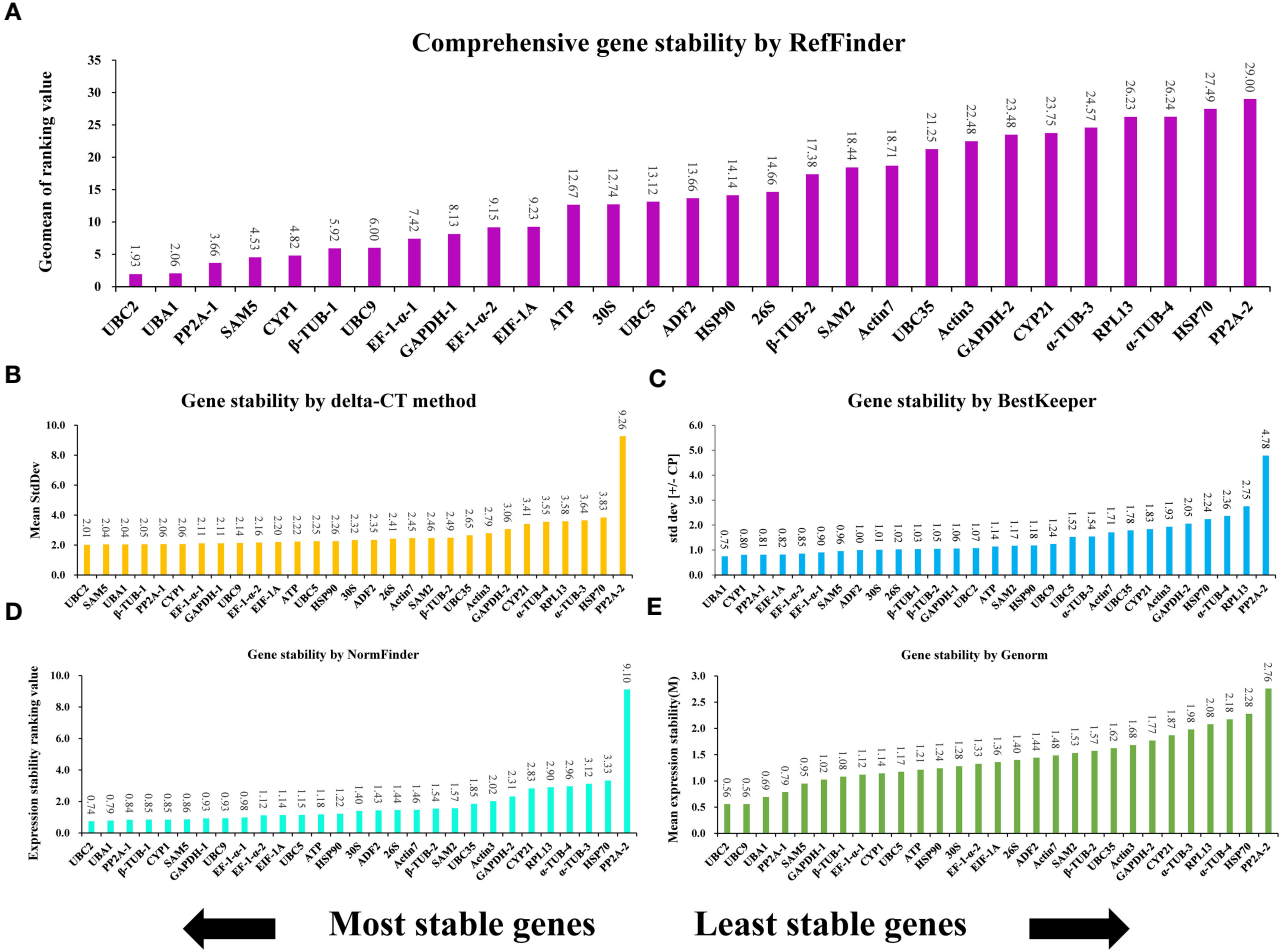
The expression stability ranking of 29 selected reference genes across samples determined vis different evaluation algorithms: **(A)** RefFinder, **(B)** delta-CT, **(C)** BestKeeper, **(D)** NormFinder, and **(E)** GeNorm.

Genes that make up the ideal reference set should be capable of stably expressing themselves in different conditions, hormone treatments, abiotic stress, and organ types (Mughal et al., 2018). To evaluate the consistency of the expression of the 29 potential genes and determine the most suitable reference gene for normalizing expression in *A. kteniophylla*, their expression levels were assessed across various treatment conditions(MeJA, ABA, SA, NaCl, Cu, Ag, Mn, Co,PEG-induced drought stress, low temperature, heat, waterlogging, wounding, and oxidative stress) via qRT-PCR (**Supplementary Table 4**). Among candidate reference genes in plant species, five software tools, namely delta-CT, BestKeeper, NormFinder, geNorm, and RefFinder were usually selected to identify appropriate reference genes. Using the delta CT method, SD values were calculated to rank the stability of the candidates, and genes with lower SD values were selected as more stable reference genes (Duan et al., 2017). According to **Figure 2B**, the results of delta CT analysis showed that *UBC2* (2.01), *SAM5* (2.04), and *UBA1* (2.04) were the most stable reference genes, while *α-TUB-3* (3.64), *HSP70* (3.83) and *PP2A-2* (9.26) were the most unstable. BestKeeper evaluate the stability of candidates by calculating the CV and SD of the Ct (cycle threshold) values of the candidate genes, and the genes with the lowest CV and SD (CV ± SD) considered to be the most stable. As depicted in **Figure 2C**, the BestKeeper analysis suggested that *UBA1* (3.49 ± 0.75), *CYP1* (4.11 ± 0.80) and *PP2A-1* (3.77 ± 0.81) were the most stable reference genes, while *PP2A-2* (17.43 ± 4.78), *RPL13* (12.94 ± 2.75) and *α-TUB-4* (7.91 ± 2.36) were found to be the least stable. GeNorm ranked the candidates according to expression stability *M* values, which are the average paired variation of a certain gene with all other candidate genes, with lower *M* values indicating more stable expression (Vandesompele et al., 2002). As can be seen in **Figure 2E**, the *UBC2* and *UBC9* genes were found to be the most stable reference genes under various treatments, with an M value of 0.56. On the contrary, *HSP70* and *PP2A-2* were the unstable gene, with *M* values of 2.28 and2.76, respectively. NormFinder ranked the potential genes according to their minimal inter- and intra-group expression variation, with lower variation values indicating higher stability of the reference genes (Andersen et al., 2004). This study examined that *UBC2* and *UBA1* genes were the most stable with the lowest ranking values of 0.74 and 0.79, while *PP2A-2* and *HSP70* were the least stable, with the ranking values at 9.10 and 3.33, respectively (**Figure 2D**). These results indicated that NormFinder and geNorm gave almost identical stability orders for the candidate genes, although minor differences were observed. In conjunction with the aforementioned four methods, the all-encompassing analysis tool, Reffinder, computes the geometric mean value of the weights assigned to the potential references, culminating in a conclusive and comprehensive ranking of said references. Through this thorough analysis, *UBC2* was identified as the most stable reference gene, with *UBA1* and *PP2A-1* following closely behind (refer to **Figure 2A**).

**Figure 3:**
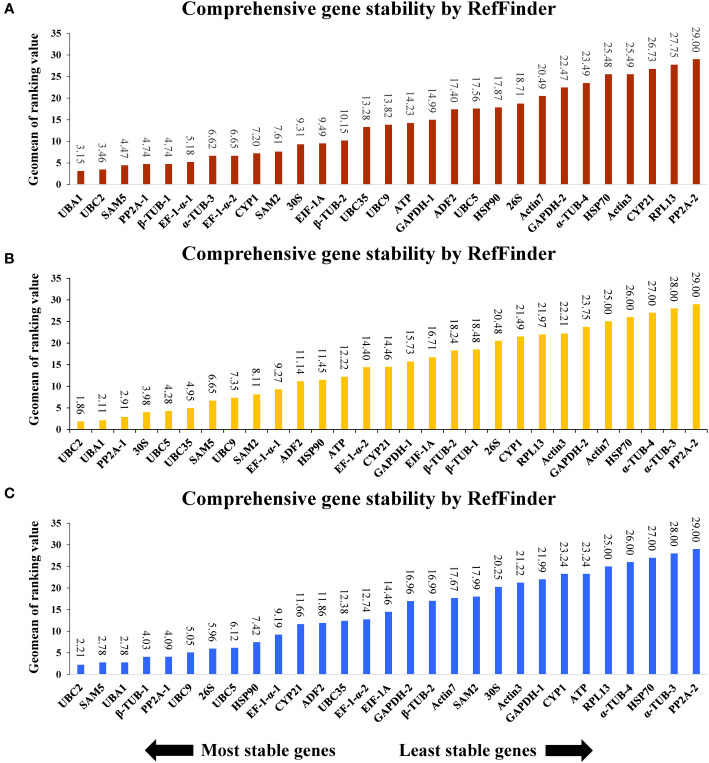
Stability rankings of 29 selected reference genes across **(A)** stress treatments, **(B)** heavy metal stress, and **(C)** hormone treatments.

To further analyze the selection of ideal references in *A. kteniophylla* under different specific conditions, all the samples treated were categorized into three groups based on exposure to heavy metal stress (Cu, Ag, Mn and Co), hormone treatment (MeJA, ABA and SA) and abiotic stress (salt, drought, cold, heat, waterlogging, injury and oxidation stress). The results depicted in **Figure 3** indicate that *UBA1* and *UBC2* exhibit the highest stability as reference genes for heavy metal stress, while *PP2A-2* displays the least stability (**Figure 3A**). As for hormone treatment and abiotic stress treatment, *UBC2* was shown to be the best gene (**Figures 3B, C**), and *PP2A-2* was confirmed as the least stable (**Figures 3B, C**).

### Application of reference genes in the anthocyanin-related genes studies of *A. kteniophylla*

**Figure 4:**
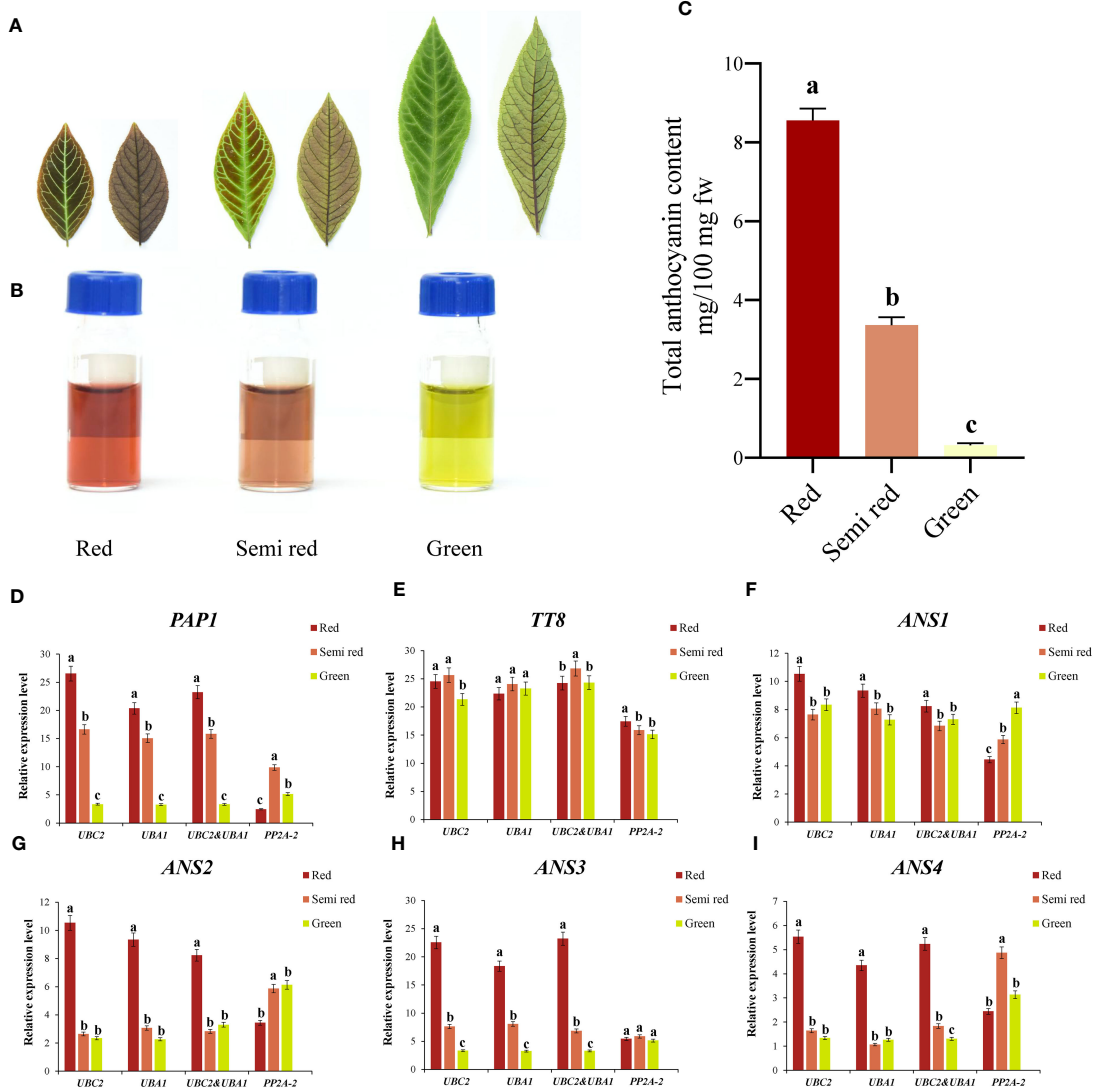
Reference genes identification and application in the anthocyanin-related genes of *A. kteniophylla*. **(A)** The leaf phenotype of red, semi red and green. (Scale bar: 2cm). **(B, C)** Relative anthocyanin content in red, semi-red, and green leaves of seedlings. **(D–I)** Relative expression levels of target gene *PAP1* **(D)**, *TT8* **(E)**, *ANS1* **(F)**, *ANS2* **(G)**, *ANS3* **(H)**, and *ANS3* **(I)** in different leaf colors were normalized with the most stable reference gene or combination and the most unstable gene. Statistically significant differences between samples are marked with different letters (*P*<0.05, Student’s t-test).

As shown in **Figures 4A–C**, *A. kteniophylla* was currently used as the ornamental plant due to its bright leaf color (including red, semi red, and green) at seedling stage, which was mainly caused by the difference in anthocyanin content. At present, numerous studies have suggested that anthocyanins were widely distributed pigments in plants, and their biosynthetic pathways and regulatory mechanisms have been widely studied in model plants and ornamental plants (Zhao and Tao, 2015). Based on comprehensive analysis via RefFinder, the top two genes (*UBC2* and *UBA1*) in A. kteniophylla were the most suitable for gene expression studies, while *PP2A-2* was not recommended as the reference gene. To determine the reliability of the reference genes selected for normalization, we selected six key genes related to anthocyanin synthesis and regulation as target genes (**Supplementary Tables 3** and **5**) (including two transcription factors: *PAP1*, *TT8* and four anthocyanin synthase genes: *ANS1*, *ANS2*, *ANS3* and *ANS4*) for normalization with the most stable reference gene or combination as well as the least stable reference gene.

According to **Figures 4D–I**, the expression of these genes tested showed similar trends when the reference genes with the most stable expression (*UBC2* and *UBA1*) were used alone or in combination under different color leaf samples, while the expression pattern of the most unstable expression reference gene (*PP2A-2*) obtained was significantly different from those of the stable references used alone or in combination. For example, when normalized with the most suitable reference genes *UBC2* or *UBA1* and their combination, the expression levels of *PAP1* was highest in the red leaves, followed by semi red leaves and lowest in green leaves. However, the expression patterns of *PAP1* were quite different from those of the optimal reference gene when normalized with the unstable gene *PP2A-2*, which *PAP1* gene showed the highest expression level in the semi red leaves, followed by green leaves and lowest in red leaves (**Figure 4D**). Therefore, this results in biased results when inappropriate reference genes are selected. In addition to confirming the significance of selecting reference genes prior to qRT-PCR, these results demonstrated that our selected genes could serve as reliable and feasible references.

Furthermore, this study found that among the six key genes that may be potentially related to anthocyanin synthesis in different leaf color samples, the transcription factor *PAP1* and structural genes *ANS2*, *ANS3* and *ANS4* had the highest expression levels in red leaves (**Figure 4**). Regardless of which reference genes and their combination (*UBC2*, *UBA1* and *UBC2* & *UBA1*). Meanwhile, the expression patterns of *PAP1* and *ANS3* were similar: red leaf > semi-red leaf > green leaf, indicating that these two genes may play a significant role in the formation and change of leaf color in *A. kteniophylla*. In contrast, the transcription factor *TT8* and structural genes *ANS1* may not have performed.

## Discussion

*A. kteniophylla*, as a significant medicinal and ornamental plant, has drawn more attention of researchers due to its showy leaf color, anticancer and other pharmacological activities. In recent years, high-throughput sequencing has become increasingly popular, RNA sequencing based on genome sequence has been widely used to detect gene expression, which provides a basis for the study of molecular biology studies of *A. kteniophylla*. Additionally, qRT-PCR was a method of high sensitivity, accuracy, and throughput for analyzing gene expression, required the ideal reference genes as a prerequisite to assure the accuracy and dependability of the results. So far, the development of stable reference genes in *A. kteniophylla* has received little attention, despite evaluating and validating reference genes in a variety of plants. Furthermore, the establishment of reference genes for stable expression in A. kteniophylla using RNA-seq datasets has not been previously reported.

### Identification of potential reference genes using RNA seq data

Recently, RNA-seq has been used to identify novel and potential reference genes for several non-model plants using expression data and assembly sequences (Li et al., 2022; Yin et al., 2021). In this study, 42 RNA-Seq datasets (unpublished) were analyzed to measure the expression levels of different genes in *A. kteniophylla* various tissues and growth years. In the present study, appropriate reference genes were selected using expression profile data to quantify genes in *A. kteniophylla*, with special attention to the application of reference genes in the study of anthocyanin synthesis and regulation related genes during the color change process of *A. kteniophylla* leaves. Mughal et al. (Mughal et al., 2018). proposed that the ideal reference gene suitable for qRT-PCR must meet the following criteria: the gene must have medium to high expression level, generally higher than the basic cell background; Meanwhile, the gene must be stably expressed across different tissues, growth stages and stress conditions, and the expression level must maintain low variation. Therefore, in order to select potential candidate reference genes, the expression level of *A. kteniophylla* genes were standardized to FPKM values using RNA seq data. These values were subsequently utilized to compute four crucial indicators, namely SD (FPKM standard deviation), MV (FPKM mean), MFC (maximum multiple change of FPKM), and CV (coefficient of variation), for assessing candidate expression stability (**Figure 1**). Meanwhile, according to numerous previous studies (Wang et al., 2021; Yang et al., 2020; Zhong et al., 2022), we tried to select some commonly used housekeeping genes as candidate genes. Finally, the 29 selected candidate genes were suitable for further screening of ideal reference genes (**Table 1**).

### Reference genes *UBC2* and *UBA1* were validated most stable

The standard qRT-PCR method was utilized to evaluate the amplification efficiency and primer specificity of all 29 candidate genes, as demonstrated in **Supplementary Figure 3** and **Table 1**. All 29 pairs of primers were found to meet the criteria for qRT-PCR experiments. To identify the optimal genes for gene expression normalization in *A. kteniophylla*, the expression levels of the 29 candidates were examined under various treatment conditions using qRT-PCR. In this study, we conducted a systematic evaluation and relatively stable reference genes using four most frequently used algorithms (Delta CT, BestKeeper, geNorm, and NormFinder) to improve the reliability of reference gene evaluation (**Figure 2**). The ranking order of the 29 candidates determined by the results of all four algorithms was reasonable but not completely consistent, mainly caused by different statistical algorithms, which have been similar findings in previous studies (Abbas et al., 2021; Li et al., 2017; Zhao et al., 2016; Zhong et al., 2022). According to previous studies, these algorithms identified mostly the same most unstable genes, but the order in which the most stable genes were sorted was different (Duan et al., 2017). Similarly, *PP2A-2* was considered the least stable genes among the four algorithms, with slightly different sequences of stable genes in this study. Therefore, we utilized the comprehensive tool RefFinder to perform final comprehensive sorting on the results of the four algorithms to improve the reliability of the results, and found that among the top stability reference genes according to RefFinder, *UBC2* was the top reference gene, followed by *UBA1* and *PP2A-1* (**Figure 2A**). Furthermore, the samples were analyzed further to select ideal reference genes in *A. kteniophylla* under three groupings of different specific conditions. **Figure 3** demonstrates that *UBA1* and *UBC2* was the most appropriate reference gene for heavy metal stress, and *UBC2* was confirmed as the most stable gene for hormone treatment and abiotic stress treatments, while *PP2A-2* was determined to be the least stable gene under all the above conditions. The finding was similar to previous research (Yin et al., 2021). The results showed that under different experimental conditions, *UBC2* and *UBA1*, with a particular emphasis on *UBC2*, were the optimal reference genes for gene expression analysis in *A. kteniophylla* across diverse experimental conditions.

Our study indicated *UBC2* was the most suitable reference gene across most samples except for heavy metal stress samples (**Figures 2** and **3**) among the 29 candidates, which may be helpful when analyzing target genes in *A. kteniophylla* using qRT-PCR. *UBC2*, as an essential component of the ubiquitin/proteasome pathway gene, has a crucial role in ubiquitination of proteins. At present, *UBC2* performed as an the optimal reference gene in *Cicer arietinum* (Castro et al., 2012), *Trifolium pratense* (Mehdi Khanlou and Van Bockstaele, 2012), *Cajanus cajan* (Sinha et al., 2015), *Corchorus capsularis* (Lin et al., 2023), and *Glycyrrhiza glabra* L (Li et al., 2020) for its highly conservative character, while exhibited low stability in *Fucus distichus* (Linardić and Braybrook, 2021). Furthermore*, UBA1* was the most stably expressed gene in heavy metal stress samples, and the second stably expressed gene in all samples. *UBA1*, as the first enzyme in the ubiquitination pathway, which encodes instructions for the production of the ubiquitin-activating enzyme E1 (Panchamia et al., 2020). It has been reported that this enzyme was necessary for the ubiquitin-proteasome system, which was one of the most important cellular processes for protein modification in plants, and involved in signaling relating to host defense (Hatfield and Vierstra, 1992; Shirsekar et al., 2010). In contrast, *PP2A-2* exhibits particularly excellent instability in all samples, despite its stable expression in other species such as *Nicotiana tabacum* (Schmidt and Delaney, 2010), *Elymus sibiricus* (Zhang et al., 2019), and *Oenanthe javanica* (Jiang et al., 2014). This discovery was similar to the results of previous studies in *Momordica charantia* (Wang et al., 2019). Previous studies have found that homologous genes exhibit similar sequences and functions, and commonly used as reference genes for gene transcription analysis (Zhou et al., 2019). As an example, as appropriate reference gene for *Fortunella crassifolia*, the *Actin* gene *ACT6*, *ACT7*, and *ACT8* were selected (Pfaffl et al., 2004). However, other research results have found opposite conclusions, such as *UBC19*, *UBC22*, and *UBC29* as homologous genes in *Isatis indigotica*, with different expression levels and relatively poor stability (Li et al., 2017). Here, We found that the homologous ubiquitin-conjugating enzyme genes (*UBC2*, *UBC5*, *UBC9*, and *UBC35*) possess completely distinct expression levels and stability characteristics (**Figure 2**). Due to this, homologous genes may show quite different expression levels and stability despite having similar sequences and functions.

### *UBC2* and *UBA1* performed well in the quantification of anthocyanin related genes in *A. kteniophylla*

In *A. kteniophella*, anthocyanins were the cause of the formation of different colors in seedling leaves (**Figures 4A–C**). Anthocyanins, as a class of flavonoids, impart color to different types of leaves, fruits, flowers, and many other tissues to prevent various biological and abiotic damage, as well as damage caused by ultraviolet or visible light (Noda et al., 2017). Additionally, anthocyanins are crucial to the cultivation of flowers, nutrition, healthcare, and the provision of medical care in human society (Yoshida et al., 2009). Molecular mechanisms underlying leaf color formation in *A. kteniophella* seedlings, however, remain poorly understood. The biosynthesis pathway of anthocyanins has been well studied in various plants, and found that *anthocyanin synthase* (*ANS*), as a key enzyme gene in anthocyanin synthesis pathway, catalyzed the conversion of leucoanthocyanidins to anthocyanins (Jun et al., 2018; Luo et al., 2018). Transcription factors bHLH and R2R3-MYB were typically involved in regulating structural genes in the flavonoid pathway, and it is particularly noteworthy that *PAP1* (R2R3-MYB family) and *TT8* (bHLH family) genes can positively regulate structural genes in the anthocyanin pathway and promote anthocyanin synthesis and accumulation (Fraser et al., 2013; Jaakola, 2013; Zhang et al., 2020). To ascertain the accuracy and reliability of the selected reference genes, a normalization procedure was conducted on the relative expression levels of target genes (namely, *PAP1*, *TT8*, *ANS1*, *ANS2*, *ANS3*, and *ANS4*) in three distinct colored leaves of *A. kteniophella* seedlings. This normalization involved the use of the two most stable reference genes, their combinations, and the least stable reference gene. The findings revealed that the employment of the most stably expressed reference genes (*UBC2* and *UBA1*) either individually or in conjunction facilitated the clear expression patterns of all target genes across the various colored leaves samples.

Even though the expression trends of each stable reference genes were consistent with those of stable reference gene combinations, there were still slight differences in expression levels. Nevertheless, the expression profile of the inappropriate reference gene (*PP2A-2*) showed complete inconsistency with the results of the stable gene and significant changes occurred. This clearly demonstrated the impact of using unstable reference genes on qRT-PCR results, and also indicated that the relative expression level of target genes varies with the selection and quantity of references. Previously, similar results have been studied in multiple species, including *Codonopsis pilosula* (Yang et al., 2020), *Scutellaria baicalensis* (Wang et al., 2021), *Aegilops tauschii* (Abbas et al., 2021), and *Dioscorea opposita* (Zhao et al., 2016). In addition, we also found that among all six key genes, the expression levels of transcription factor *PAP1* and structural genes *ANS2*, *ANS3*, and *ANS4* were highest in red leaves (**Figure 4**), especially the expression profiles of *PAP1* and *ANS3* were red leaves>semi red leaves>green leaves, suggesting that these two genes may play a significant role in the anthocyanin biosynthesis pathway in embryonic leaves. Taking these findings together, they highlight the fundamental significance of selecting ideal reference genes before performing qRT-PCR.

## Conclusion

Here, we systematically identified and screened ideal reference genes under diverse experimental conditions for the first time, which were used for qRT-PCR analysis of target gene expression in *Ardisia* genus plant, *A. kteniophella*. From our results, two stably expressing genes, *UBC2* and *UBA1*, were identified as appropriate for accurately normalizing target gene expression in *A. kteniophella* across all experimental conditions. In addition, *UBC2* and *UBA1* have also been successfully used for gene expression analysis in the biological study of color difference in the *A. kteniophella* leaves, and found that *PAP1* and *ANS3* may play a critical role in the formation and change of leaf color in *A. kteniphella* for the first time. These findings not only make a significant contribution to functional genomics analysis for *A. kteniphella*, but they also lay a good foundation for the study of molecular mechanism of biological function in *A. kteniphella* in the future. Besides, the stable references of this study provide reference guidelines for other species that have not validated qRT-PCR analysis reference genes.

## Data availability statement

The datasets presented in this study can be found in online repositories. The names of the repository/repositories and accession number(s) can be found below: BioProject, PRJNA1026808.

## Author contributions

WW: Conceptualization, Formal Analysis, Methodology, Validation, Writing – original draft, Writing – review & editing. XZ: Data curation, Formal Analysis, Methodology, Software, Visualization, Writing – review & editing. XXX: Data curation, Formal Analysis, Methodology, Validation, Writing – review & editing. XCX: Data curation, Methodology, Software, Visualization, Writing – review & editing. LF: Data curation, Formal Analysis, Investigation, Visualization, Writing – review & editing. HC: Conceptualization, Formal Analysis, Funding acquisition, Resources, Writing – review & editing.

## Supplementary material

Supplementary Figure 1 Primers were validated by dissolving amplicons of candidate references on agarose gel.

Supplementary Figure 2 The melting curves of the 29 amplicons in A*. kteniophylla* showed a single peak for each ample.

Supplementary Figure 3 Analyses of candidate references and target genes for amplification efficiency (*E*) and regression coefficient (*R^2^*). The standard curve equation is obtained by taking the logarithmic value of the corresponding gene concentration diluted multiple as the x-axis and the Ct value as the y-axis.

## References

[B1] Abbas A. Yu H. Li X. Cui H. Chen J. Huang P. (2021). Selection and validation of reference genes for RT-qPCR analysis in *Aegilops tauschii* (coss.) under different abiotic stresses. Int. J. Mol. Sci. 22 (20), 2011017. doi: 10.3390/ijms222011017 PMC8541341 34681677

[B2] An J. P. Wang X. F. Zhang X. W. Xu H. F. Bi S. Q. You C. X.. (2020). An apple MYB transcription factor regulates cold tolerance and anthocyanin accumulation and undergoes MIEL1-mediated degradation. Plant Biotechnol. J. 18, 337–353. doi: 10.1111/pbi.13201 31250952 PMC6953192

[B3] Andersen C. L. Jensen J. L. Ørntoft T. F. (2004). Normalization of real-time quantitative reverse transcription-PCR data: a model-based variance estimation approach to identify genes suited for normalization, applied to bladder and colon cancer data sets. Cancer Res. 64, 5245–5250. doi: 10.1158/0008-5472.CAN-04-0496 15289330

[B4] Castro P. Román B. Rubio J. Die J. V. (2012). Selection of reference genes for expression studies in *Cicer arietinum* L.: analysis of cyp81E3 gene expression against *Ascochyta rabiei* . Mol. Breed 29, 261–274. doi: 10.1007/s11032-010-9544-8

[B5] Chen C. Chen H. Zhang Y. Thomas H. R. Frank M. H. He Y.. (2020). TBtools: an integrative toolkit developed for interactive analyses of big biological data. Mol. Plant 13, 1194–1202. doi: 10.1016/j.molp.2020.06.009 32585190

[B6] de Magalhães J. P. Finch C. E. Janssens G. (2010). Next-generation sequencing in aging research: emerging applications, problems, pitfalls and possible solutions. Ageing Res. Rev. 9, 315–323. doi: 10.1016/j.arr.2009.10.006 19900591 PMC2878865

[B7] Duan M. Wang J. Zhang X. Yang H. Wang H. Qiu Y.. (2017). Identification of optimal reference genes for expression analysis in radish (*Raphanus sativus* L.) and its relatives based on expression stability. Front. Plant Sci. 8. doi: 10.3389/fpls.2017.01605 PMC5605625 28966627

[B8] Fraser L. G. Seal A. G. Montefiori M. McGhie T. K. Tsang G. K. Datson P. M.. (2013). An R2R3 MYB transcription factor determines red petal colour in an *Actinidia* (kiwifruit) hybrid population. BMC Genomics 14, 28. doi: 10.1186/1471-2164-14-28 23324587 PMC3618344

[B9] Gao M. Liu Y. Ma X. Shuai Q. Gai J. Li Y. (2017). Evaluation of reference genes for normalization of gene expression using quantitative RT-PCR under *aluminum, cadmium*, and heat stresses in soybean. PloS One 12, e0168965. doi: 10.1371/journal.pone.0168965 28046130 PMC5207429

[B10] González-Agüero M. García-Rojas M. Di Genova A. Correa J. Maass A. Orellana A.. (2013). Identification of two putative reference genes from grapevine suitable for gene expression analysis in berry and related tissues derived from RNA-Seq data. BMC Genomics 14, 878. doi: 10.1186/1471-2164-14-878 24330674 PMC3878734

[B11] Gou T. Yang L. Hu W. Chen X. Zhu Y. Guo J.. (2020). Silicon improves the growth of cucumber under excess nitrate stress by enhancing nitrogen assimilation and chlorophyll synthesis. Plant Physiol. Biochem. 152, 53–61. doi: 10.1016/j.plaphy.2020.04.031 32388420

[B12] Guan Y. F. Song X. Qiu M. H. Luo S. H. Wang B. J. Hung. V.. (2016). Bioassay-guided isolation and structural modification of the anti-TB resorcinols from *Ardisia gigantifolia.* Chem. Biol. Drug Des. 88, 293–301. doi: 10.1111/cbdd.12756 26992112

[B13] Hatfield P. M. Vierstra R. D. (1992). Multiple forms of ubiquitin-activating enzyme E1 from wheat. Identification of an essential cysteine by in *vitro* mutagenesis. J. Biol. Chem. 267, 14799–14803. doi: 10.1016/S0021-9258(18)42110-2 1634524

[B14] Huang G. H. Hu C. M. Gang H. (2017). Rediscovery of *Ardisia gigantifolia* Stapf and the reinstatement of *A. kteniophylla* A. DC. (*Primulaceae*). Nordic J. Bot. 35, 628–632. doi: 10.1111/njb.01672

[B15] Jaakola L. (2013). New insights into the regulation of anthocyanin biosynthesis in fruits. Trends Plant Sci. 18, 477–483. doi: 10.1016/j.tplants.2013.06.003 23870661

[B16] Jiang Q. Wang F. Li M. Y. Ma J. Tan G. F. Xiong A. S. (2014). Selection of suitable reference genes for qPCR normalization under abiotic stresses in *Oenanthe javanica* (BI.) DC. PloS One 9, e92262. doi: 10.1371/journal.pone.0092262 24651080 PMC3961309

[B17] Jun J. H. Xiao X. Rao X. Dixon R. A. (2018). Proanthocyanidin subunit composition determined by functionally diverged dioxygenases. Nat. Plants 4, 1034–1043. doi: 10.1038/s41477-018-0292-9 30478357

[B18] Kudo T. Sasaki Y. Terashima S. Matsuda-Imai N. Takano T. Saito M.. (2016). Identification of reference genes for quantitative expression analysis using large-scale RNA-seq data of *Arabidopsis thaliana* and model crop plants. Genes Genet. Syst. 91, 111–125. doi: 10.1266/ggs.15-00065 27040147

[B19] Kumar V. Sharma R. Trivedi P. C. Vyas G. K. Khandelwal V. (2011). Traditional and novel references towards systematic normalization of qRT-PCR data in plants. Aust. J. Crop Sci. 5, 1455–1468. doi: 10.1016/j.fcr.2011.04.016

[B20] Li J. Han X. Wang C. Qi W. Zhang W. Tang L.. (2017). Validation of suitable reference genes for RT-qPCR data in *Achyranthes bidentata* blume under different experimental conditions. Front. Plant Sci. 8. doi: 10.3389/fpls.2017.00776 PMC5432617 28559905

[B21] Li Y. Liang X. Zhou X. Wu Z. Yuan L. Wang Y.. (2020). Selection of reference genes for qRT-PCR analysis in medicinal plant *Glycyrrhiza* under abiotic stresses and hormonal treatments. Plants (Basel) 9 (11), 111441. doi: 10.3390/plants9111441 PMC7692165 33114570

[B22] Li G. Ma J. Yin J. Guo F. Xi K. Yang P.. (2022). Identification of reference genes for reverse transcription-quantitative PCR analysis of ginger under abiotic stress and for postharvest biology studies. Front. Plant Sci. 13. doi: 10.3389/fpls.2022.893495 PMC9207462 35734245

[B23] Li T. Wang J. Lu M. Zhang T. Qu X. Wang Z. (2017). Selection and validation of appropriate reference genes for qRT-PCR analysis in *Isatis indigotica* fort. Front. Plant Sci. 8. doi: 10.3389/fpls.2017.01139 PMC5487591 28702046

[B24] Lin Y. Liu G. Rao Y. Wang B. Tian R. Tan Y.. (2023). Identification and validation of reference genes for qRT-PCR analyses under different experimental conditions in *Allium wallichii* . J. Plant Physiol. 281, 153925. doi: 10.1016/j.jplph.2023.153925 36657231

[B25] Linardić M. Braybrook S. A. (2021). Identification and selection of optimal reference genes for qPCR-based gene expression analysis in *Fucus distichus* under various abiotic stresses. PloS One 16, e0233249. doi: 10.1371/journal.pone.0233249 33909633 PMC8081170

[B26] Liu Y. Lin-Wang K. Espley R. V. Wang L. Yang H. Yu B.. (2016). Functional diversification of the potato R2R3 MYB anthocyanin activators AN1, MYBA1, and MYB113 and their interaction with basic helix-loop-helix cofactors. J. Exp. Bot. 67, 2159–2176. doi: 10.1093/jxb/erw014 26884602 PMC4809278

[B27] Luo H. Dai C. Li Y. Feng J. Liu Z. Kang C. (2018). Reduced Anthocyanins in Petioles codes for a GST anthocyanin transporter that is essential for the foliage and fruit coloration in strawberry. J. Exp. Bot. 69, 2595–2608. doi: 10.1093/jxb/ery096 29538703 PMC5920330

[B28] Luo M. Gao Z. Li H. Li Q. Zhang C. Xu W.. (2018). Selection of reference genes for miRNA qRT-PCR under abiotic stress in grapevine. Sci. Rep. 8, 4444. doi: 10.1038/s41598-018-22743-6 29535408 PMC5849727

[B29] Ma R. Xu S. Zhao Y. Xia B. Wang R. (2016). Selection and validation of appropriate reference genes for quantitative real-time PCR analysis of gene expression in *Lycoris aurea* . Front. Plant Sci. 7. doi: 10.3389/fpls.2016.00536 PMC4843812 27200013

[B30] Mehdi Khanlou K. Van Bockstaele E. (2012). A critique of widely used normalization software tools and an alternative method to identify reliable reference genes in red clover (*Trifolium pratense* L.). Planta 236, 1381–1393. doi: 10.1007/s00425-012-1682-2 22718310

[B31] Mitteer D. R. Greer B. D. Fisher W. W. Cohrs V. L. (2018). Teaching behavior technicians to create publication-quality, single-case design graphs in graphpad prism 7. J. Appl. Behav. Anal. 51, 998–1010. doi: 10.1002/jaba.483 29971776 PMC6188791

[B32] Mu L. H. Gu Y. J. Wang L. H. Ma B. P. Lu L. Liu P. (2015). Biotransformation on the triterpenoid saponin of *Ardisia gigantifolia* by *Aspergillus avenaceus* AS 3.4454. J. Asian Nat. Prod Res. 17, 40–46. doi: 10.1080/10286020.2014.958997 25494647

[B33] Mu L. H. Wang Y. N. Wang D. X. Zhang J. Liu L. Dong X. Z.. (2017). AG36 inhibits human breast cancer cells proliferation by promotion of apoptosis *in vitro* and *in vivo* . Front. Pharmacol. 8. doi: 10.3389/fphar.2017.00015 PMC5266696 28184196

[B34] Mu L. H. Wei N. Y. Liu P. (2012). Cytotoxic triterpenoid saponins from *Ardisia gigantifolia* . Planta Med. 78, 617–621. doi: 10.1055/s-0031-1298254 22314414

[B35] Mughal B. B. Leemans M. Spirhanzlova P. Demeneix B. Fini,. J. B. (2018). Reference gene identification and validation for quantitative real-time PCR studies in developing *Xenopus laevis* . Sci. Rep. 8, 496. doi: 10.1038/s41598-017-18684-1 29323148 PMC5764961

[B36] Noda N. Yoshioka S. Kishimoto S. Nakayama M. Douzono M. Tanaka Y.. (2017). Generation of blue chrysanthemums by anthocyanin B-ring hydroxylation and glucosylation and its coloration mechanism. Sci. Adv. 3, e1602785. doi: 10.1126/sciadv.1602785 28782017 PMC5529055

[B37] Nolan T. Hands R. E. Bustin S. A. (2006). Quantification of mRNA using real-time RT-PCR. Nat. Protoc. 1, 1559–1582. doi: 10.1038/nprot.2006.236 17406449

[B38] Panchamia B. Raimalani V. Prashar V. Kumar M. Ratna Prabha C. (2020). Structural and functional characterisation of the domains of ubiquitin-activating enzyme (E1) of Saccharomyces cerevisiae. Cell Biochem. Biophys. 78, 309–319. doi: 10.1007/s12013-020-00924-3 32583128

[B39] Pfaffl M. W. Tichopad A. Prgomet C. Neuvians T. P. (2004). Determination of stable housekeeping genes, differentially regulated target genes and sample integrity: BestKeeper–Excel-based tool using pair-wise correlations. Biotechnol. Lett. 26, 509–515. doi: 10.1023/b:bile.0000019559.84305.47 15127793

[B40] Rhinn H. Marchand-Leroux C. Croci N. Plotkine M. Scherman D. Escriou V. (2008). Housekeeping while brain’s storming validation of normalizing factors for gene expression studies in a murine model of traumatic brain injury. BMC Mol. Biol. 9, 62. doi: 10.1186/1471-2199-9-62 18611280 PMC2500043

[B41] Schmidt G. W. Delaney S. K. (2010). Stable internal reference genes for normalization of real-time RT-PCR in tobacco (*Nicotiana tabacum*) during development and abiotic stress. Mol. Genet. Genomics 283, 233–241. doi: 10.1007/s00438-010-0511-1 20098998

[B42] Shirsekar G. Dai L. Hu Y. Wang X. Zeng L. Wang G. L. (2010). Role of ubiquitination in plant innate immunity and pathogen virulence. J. Plant Biol. 53, 10–18. doi: 10.1007/s12374-009-9087-x

[B43] Silver N. Best S. Jiang J. Thein S. L. (2006). Selection of housekeeping genes for gene expression studies in human reticulocytes using real-time PCR. BMC Mol. Biol. 7, 33. doi: 10.1186/1471-2199-7-33 17026756 PMC1609175

[B44] Sinha P. Saxena R. K. Singh V. K. Krishnamurthy L. Varshney R. K. (2015). Selection and validation of housekeeping genes as reference for gene expression studies in pigeonpea (*Cajanus cajan*) under heat and salt stress conditions. Front. Plant Sci. 6. doi: 10.3389/fpls.2015.01071 PMC4865767 27242803

[B45] Stone J. D. Storchova H. (2015). The application of RNA-seq to the comprehensive analysis of plant mitochondrial transcriptomes. Mol. Genet. Genomics 290, 1–9. doi: 10.1007/s00438-014-0905-6 25182379

[B46] Tian L. Xi Gu Ri G. Yu J. Qu S. Xie Q. Shama R.. (2023). *Ardisia gigantifolia* stapf (*Primulaceae*): A review of ethnobotany, phytochemistry, pharmacology, clinical application, and toxicity. J. Ethnopharmacol. 305, 116079. doi: 10.1016/j.jep.2022.116079 36603784

[B47] Udvardi M. K. Czechowski T. Scheible W. R. (2008). Eleven golden rules of quantitative RT-PCR. Plant Cell 20, 1736–1737. doi: 10.1105/tpc.108.061143 18664613 PMC2518243

[B48] Vandesompele J. De Preter K. Pattyn F. Poppe B. Van Roy N. De Paepe A.. (2002). Accurate normalization of real-time quantitative RT-PCR data by geometric averaging of multiple internal control genes. Genome Biol. 3, Research0034. doi: 10.1186/gb-2002-3-7-research0034 12184808 PMC126239

[B49] Wang C. Cui H. M. Huang T. H. Liu T. K. Hou X. L. Li Y. (2016). Identification and validation of reference genes for RT-qPCR analysis in non-heading Chinese cabbage flowers. Front. Plant Sci. 7. doi: 10.3389/fpls.2016.00811 PMC4901065 27375663

[B50] Wang Q. Guo C. Yang S. Zhong Q. Tian J. (2023). Screening and verification of reference genes for analysis of gene expression in garlic (*Allium sativum* L.) under Cold and Drought Stress. Plants (Basel) 12 (4), 763. doi: 10.3390/plants12040763 36840111 PMC9963267

[B51] Wang W. Hu S. Cao Y. Chen R. Wang Z. Cao X. (2021). Selection and evaluation of reference genes for qRT-PCR of *Scutellaria baicalensis* Georgi under different experimental conditions. Mol. Biol. Rep. 48, 1115–1126. doi: 10.1007/s11033-021-06153-y 33511512 PMC7842394

[B52] Wang Z. Xu J. Liu Y. Chen J. Lin H. Huang Y.. (2019). Selection and validation of appropriate reference genes for real-time quantitative PCR analysis in *Momordica charantia* . Phytochemistry 164, 1–11. doi: 10.1016/j.phytochem.2019.04.010 31054374

[B53] Xie F. Xiao P. Chen D. Xu L. Zhang B. (2012). miRDeepFinder: a miRNA analysis tool for deep sequencing of plant small RNAs. Plant Mol. Biol. 80 (1), 75–84. doi: 10.1007/s11103-012-9885-2 22290409

[B54] Xu Z. S. Yang Q. Q. Feng K. Yu X. Xiong A. S. (2020). *DcMYB113*, a root-specific R2R3-MYB, conditions anthocyanin biosynthesis and modification in carrot. Plant Biotechnol. J. 18, 1585–1597. doi: 10.1111/pbi.13325 31910327 PMC7292547

[B55] Yang J. Yang X. Kuang Z. Li B. Lu X. Cao X.. (2020). Selection of suitable reference genes for qRT-PCR expression analysis of *Codonopsis pilosula* under different experimental conditions. Mol. Biol. Rep. 47, 4169–4181. doi: 10.1007/s11033-020-05501-8 32410139

[B56] Yim A. K. Wong J. W. Ku Y. S. Qin H. Chan T. F. Lam H. M. (2015). Using RNA-seq data to evaluate reference genes suitable for gene expression studies in soybean. PloS One 10, e0136343. doi: 10.1371/journal.pone.0136343 26348924 PMC4562714

[B57] Yin J. Hou L. Jiang X. Yang J. He Y. Zhou X.. (2021). Identification and validation of reference genes for quantitative real-time PCR studies in alligatorweed (*Alternanthera philoxeroides*). Weed Sci. 69, 404–411. doi: 10.1017/wsc.2021.32

[B58] Yoshida K. Mori M. Kondo T. (2009). Blue flower color development by anthocyanins: from chemical structure to cell physiology. Nat. Prod Rep. 26, 884–915. doi: 10.1039/b800165k 19554240

[B59] Zhang J. Lei Y. Wang B. Li S. Yu S. Wang Y.. (2020). The high-quality genome of diploid strawberry (*Fragaria nilgerrensis*) provides new insights into anthocyanin accumulation. Plant Biotechnol. J. 18, 1908–1924. doi: 10.1111/pbi.13351 32003918 PMC7415782

[B60] Zhang J. Xie W. Yu X. Zhang Z. Zhao Y. Wang N.. (2019). Selection of suitable reference genes for RT-qPCR gene expression analysis in siberian wild rye (*Elymus sibiricus*) under different experimental conditions. Genes (Basel) 10 (6), 451. doi: 10.3390/genes10060451 31200580 PMC6627066

[B61] Zhao D. Tao J. (2015). Recent advances on the development and regulation of flower color in ornamental plants. Front. Plant Sci. 6. doi: 10.3389/fpls.2015.00261 PMC4410614 25964787

[B62] Zhao B. Xiong C. Wu L. Xiang L. Chen S. (2021). DNA barcoding coupled with high resolution melting for rapid identification of *Ardisia gigantifolia* and its toxic adulterants. Biotechnol. Biotechnol. Equip. 35, 641–649. doi: 10.1080/13102818.2021.1885993

[B63] Zhao X. Zhang X. Guo X. Li S. Han L. Song Z.. (2016). Identification and Validation of Reference Genes for qRT-PCR Studies of Gene Expression in *Dioscorea opposita* . BioMed. Res. Int. 2016, 3089584. doi: 10.1155/2016/3089584 27314014 PMC4899605

[B64] Zhong Y. Gai Y. Gao J. Nie W. Bao Z. Wang W.. (2022). Selection and validation of reference genes for quantitative real-time PCR normalization in *Psoralea corylifolia* (Babchi) under various abiotic stress. J. Plant Physiol. 274, 153722. doi: 10.1016/j.jplph.2022.153722 35605384

[B65] Zhou Z. Cong P. Tian Y. Zhu Y. (2017). Using RNA-seq data to select reference genes for normalizing gene expression in apple roots. PloS One 12, e0185288. doi: 10.1371/journal.pone.0185288 28934340 PMC5608369

[B66] Zhou W. Wang S. Yang L. Sun Y. Zhang Q. Li B.. (2019). Reference genes for qRT-PCR normalisation in different tissues, developmental stages, and stress conditions of *Hypericum perforatum* . PeerJ 7, e7133. doi: 10.7717/peerj.7133 31259099 PMC6589333

