# Supplementary figures and images for: Systematic identification of reference genes for qRT-PCR of Ardisia kteniophylla A. DC under different experimental conditions and for anthocyanin-related genes studies
Source: Front Plant Sci. 2023 Nov 2;14:1284007. doi: 10.3389/fpls.2023.1284007 (PMC10656778; doi:10.3389/fpls.2023.1284007)

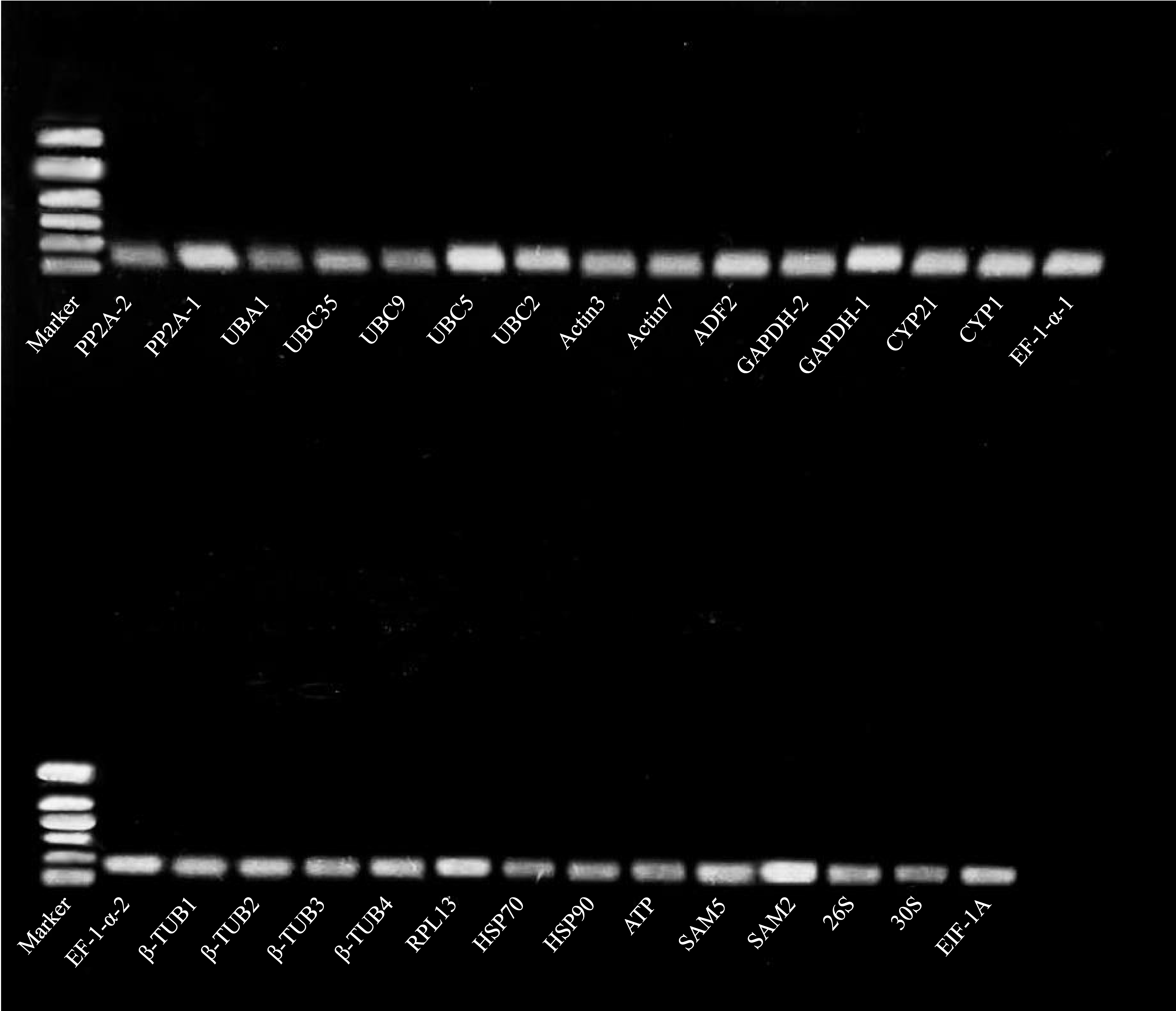

Supplement: Supplementary Figure 1 — Primers were validated by dissolving amplicons of candidate references on agarose gel. [file DataSheet_1.zip › Additional files/Figure S1.tif]
